# The Respiratory Burst Oxidase Homolog D (RbohD) Cell and Tissue Distribution in Potato–Potato Virus Y (PVY^NTN^) Hypersensitive and Susceptible Reactions

**DOI:** 10.3390/ijms20112741

**Published:** 2019-06-04

**Authors:** Katarzyna Otulak-Kozieł, Edmund Kozieł, Rodrigo A. Valverde

**Affiliations:** 1 Department of Botany, Faculty of Agriculture and Biology, Warsaw University of Life Sciences—SGGW, Nowoursynowska Street 159, 02-776 Warsaw, Poland; edmund_koziel@sggw.pl; 2 Department of Plant Pathology and Crop Physiology, Louisiana State University Agricultural Center, Baton Rouge, LA 70803, USA; RValverde@agcenter.lsu.edu

**Keywords:** hypersensitive response, potato virus Y, immunolocalization, ultrastructure, respiratory burst oxidase homolog D, *Solanum tuberosum*

## Abstract

The respiratory burst oxidase homolog D (RbohD) acts as a central driving force of reactive oxygen species signaling in plant cells by integrating many different signal transduction pathways in plants, including incompatible interactions with pathogens. This study demonstrated the localization and distribution of RbohD in two types of potato–potato virus Y (PVY) interactions: Compatible and incompatible (resistant). The results indicated a statistically significant induction of the RbohD antigen signal in both interaction types. In the hypersensitive response (resistant reaction) of potato with a high level of resistance to the potato tuber necrotic strain of PVY (PVY^NTN^), RbohD localization followed by hydrogen peroxide (H_2_O_2_) detection was concentrated in the apoplast. In contrast, in the hypersensitive response of potato with a low resistance level to PVY^NTN^, the distribution of RbohD was concentrated more in the plant cell organelles than in the apoplast, resulting in the virus particles being present outside the inoculation area. Moreover, when compared to mock-inoculated plants and to the hypersensitive response, the PVY^NTN^-compatible potato interaction triggered high induction in the RbohD distribution, which was associated with necrotization. Our findings indicated that RbohD and hydrogen peroxide deposition was associated with the hypersensitive response, and both were detected in the vascular tissues and chloroplasts. These results suggest that the RbohD distribution is actively dependent on different types of PVY ^NTN^-potato plant interactions. Additionally, the RbohD may be involved in the PVY^NTN^ tissue limitation during the hypersensitive response, and it could be an active component of the systemic signal transduction in the susceptible host reaction.

## 1. Introduction

Potato virus Y (PVY, *Potyvirus*) is considered the fifth most economically important plant virus [1]. PVY infects not only food crop plants, but also many ornamental plant species. The potato tuber necrotic strain of PVY (PVY^NTN^) causes symptoms that consist of vein necrosis, systemic mottle, and necrotic ringspots, which dramatically decrease the crop quality [2]. Host response to PVY^NTN^ infection, like with other viruses, varies depending on the host resistance level. Plant virus-interactions can be categorized into two groups: Compatible and incompatible [3,4]. In the first type, viruses are able to replicate, cause dynamic changes of the plant cell ultrastructure, and are transported systemically within the host tissues [5]. Whereas, in an incompatible interaction, the virus presence triggers localized death of cells and tissues, limiting the spread of the virus. Nevertheless, the blockage of the virus spread is not always complete [5]. In contrast to bacteria or fungal pathogens, PVY enters the plant tissues and is immediately associated with host cells, causing organelle alterations before the plant is able to induce a defense response [5]. Besides ultrastructural effects, the host metabolism is significantly altered after PVY infection [6]. Peroxidase activity, levels of respiration, and photosynthetic rates are also affected by PVY [6]. Virus infection and other pathogens result in oxidative stress processes named oxidative burst or accumulation of reactive oxygen species (ROS). ROS are involved in different pathways of plant–pathogen interactions [7,8,9]. For example, localized virus inhibition by oxidative stress or defense signaling has been demonstrated on various plant–pathogen interactions [10,11]. The first evidence for the role of oxidative stress and ROS in resistant plants was reported by Doke and Ohashi [12]. They postulated that there was an early oxidative burst in the Tobacco mosaic virus (TMV)—*Nicotiana tabacum* interactions.

Plant NADPH oxidases, named respiratory burst oxidase homologues (RBOHs), have been demonstrated to be a major source of ROS during plant-microbe interaction [13,14]. RBOHs are members of a small family of 10 highly conserved proteins in the plant model *Arabidopsis thaliana* [15]. RBOH homologues are membrane proteins with six transmembrane domains [16]. The C-terminal region has been characterized as an NADPH and FAD- binding domains [16]. The N-terminal region has two calcium-binding domains (EF-hand motives) and a phosphorylation domain. RBOH functionally catalyzes the superoxide anion formation process [17]. Superoxide anion radicals form hydrogen peroxide (H_2_O_2_) spontaneously by dismutation [18]. The function of NADPH oxidases is associated with developmental processes such as root hair formation, pollen tip growth, and seed ripening, among others [19,20]. Interestingly, RBOH also mediates multiple processes to balance cellular homeostasis under stress conditions [15]. The most highly expressed NADPH oxidase enzyme in pathogen response processes is respiratory burst oxidase homologue D (RbohD) [16]. Moreover, RbohD-dependent ROS has been shown to be involved in the plant defense reaction to pathogens associated with the hypersensitive response [16,21,22]. The functions of RbohD in plant–pathogen interactions have been analyzed in various plant species using knockout mutants. The RbohD and RbohF isoforms are responsible for ROS burst in reaction to pathogen associated molecular patterns [22,23]. As the principal ROS producer, RbohD has been investigated in infections of plants by the oomycete *Phytophtora* sp. or the fungus *Botrytis cinerea* and in many host–pathogen interactions that induce SAR (systemic acquired resistance) or HR (hypersensitive response) reactions [24,25,26]. Independently of the biological interaction, convergent tendencies were observed—ROS accumulation simultaneously with RbohD transcript and protein up-regulation. It has been shown that after inoculation of *Cucumis sativus* or *Cucurbita pepo* with Zucchini yellow mosaic virus (ZYMV), the host antioxidative metabolism was affected by enzymes such as proline oxidase (POX), catalase (CAT), or glutathione (GSH) [27]. Moreover, ZYMV-enhanced peroxidases via formation of ROS were associated with the development of mosaic and yellowing symptoms [27].

In this investigation, we carried out experiments to test if a fundamental ROS-producer such as RbohD was involved in PVY-potato interactions after mechanical inoculations. We investigated the subcellular localization and distribution of RbohD in compatible and incompatible PVY-potato interactions using plants with different levels of resistance to PVY.

## 2. Results

### 2.1. Immunofluorescence Localization of RbohD Protein in Compatible and Incompatible Interactions

**Figure 1:**
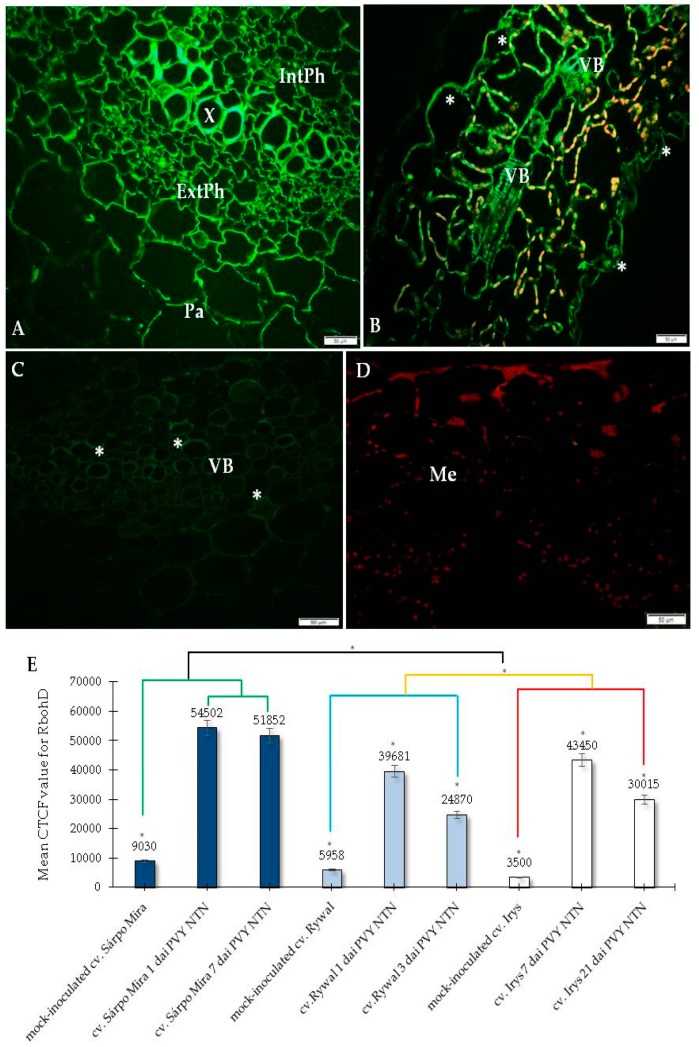
Fluorescence detection of RbohD protein in an incompatible interaction of potato Sárpo Mira plants with potato virus Y (PVY) ^NTN^ (**A**–**D**) and the assessment of the quantitative fluorescence signal in all interactions (**E**). (**A**) The green fluorescence signal of RbohD (*) in xylem, external and internal phloem, and parenchyma cells 1 day after PVY^NTN^ inoculation. (**B**) The green fluorescence signal of RbohD (*) in the epidermis (*), also with stomata and vascular bundles 7 days after PVY ^NTN^ inoculation. (**C**) RbohD signal (*) in a vascular bundle of mock-inoculated Sárpo Mira leaf. (**D**) Control—lack of green fluorescence signal in hypersensitive response when primary antibodies were omitted. (**E**) The assessment of the quantitative fluorescence signal of RbohD was conducted by using the corrected total cell fluorescence method (CTCF) in combination with ANOVA. The dark blue charts represent mock-inoculated and PVY-inoculated Sárpo Mira (resistant) potato plants before the hypersensitive reaction (HR) symptoms—1 day after inoculation (dai) and when HR symptoms developed—7 dai. The light blue charts represent mock-inoculated and PVY ^NTN^-inoculated Rywal (less resistant) before the HR symptoms—1 dai and when HR symptoms developed—3 dai. The white charts represent control plants (mock-inoculated) and also PVY ^NTN^-inoculated cv. Irys (susceptible) potato plants in 7 and 21 dai. Mean values of CTCF were evaluated at the *p* <0.05 level of significance using a post-hoc Tukey HSD test. Statistically significant values in different days after mock or viral inoculation for the cultivars are marked by asterisks (*) above the mean value of the CTCF on each chart bar. Statistically significant values between cultivars are marked by color brackets with asterisks (*). An orange bracket with one asterisk (*) indicates a statistically significant difference between mock-inoculated, virus inoculated (3dai) cv Rywal plants (blue bracket), and the mock-inoculated virus (21dai) cy Irys plants (red bracket). A black bracket with an asterisk (*) indicates a significant statistical difference between cv Sárpo Mira (green bracket) and the other two cultivars, Rywal and also Irys. Bar = 50 µm. Ep-epidermis, ExtPh—external phloem, IntPh—internal phloem, Me—mesophyll, Pa–parechnyma, VB—vascular bundle, X-xylem.

**Figure 2:**
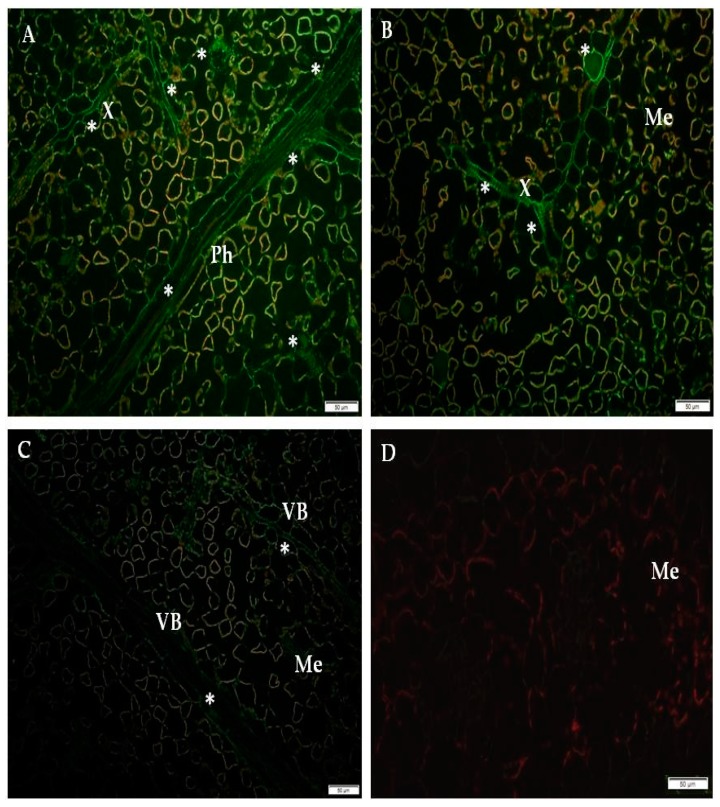
Immunofluorescence of RbohD protein localization in an incompatible interaction of potato Rywal plants with PVY ^NTN^. (**A**) Signal of RbohD (*) in phloem and xylem cells 1 day after PVY^NTN^-inoculation. (**B**) RbohD signal (*) in xylem tracheary elements and a single necrotizing mesophyll cell. (**C**) RbohD signal (*) in the vascular bundle of mock-inoculated potato Rywal. (**D**) Control-lack of green fluorescence signal in hypersensitive response potato Rywal when primary antibodies were omitted. Bar 50 µm. Me—mesophyll, Ph—phloem, X—xylem, VB—vascular bundle.

**Figure 3:**
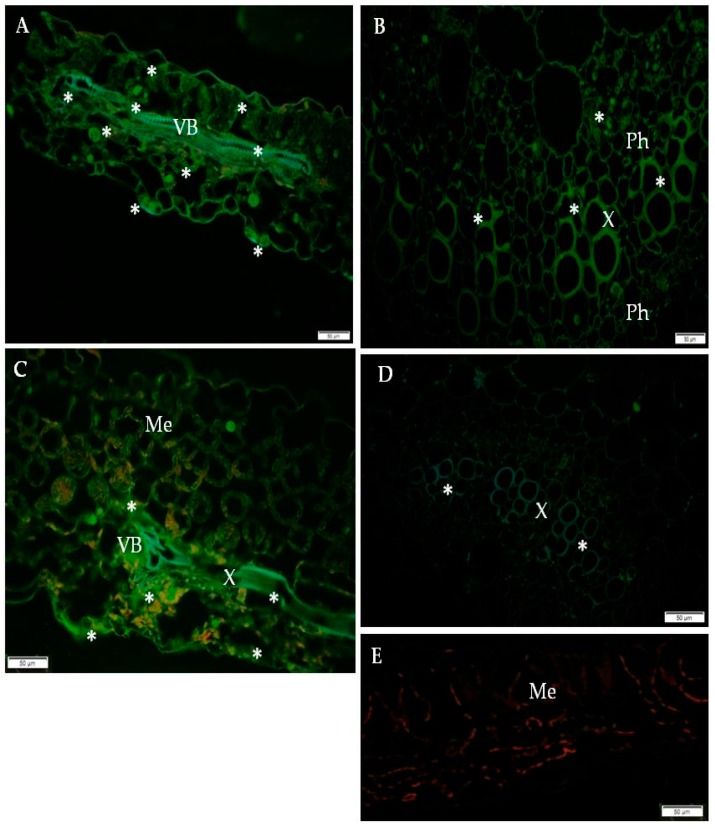
Fluorescence detection of RbohD protein in a compatible interaction of potato Irys plants with PVY ^NTN^. (**A**) RbohD signal (*) in vascular bundle, stomata in epidermis and mesophyll 7 days after PVY^NTN^-inoculation. (**B**) Green fluorescence of RbohD in vascular tissues 7 dai. (**C**) Green fluorescence of RbohD in necrotizing area of the vascular bundle with the epidermis 21 dai. (**D**) RbohD signal in xylem tracheary elements in mock inoculated potato Irys plant. (**E**) Lack of green fluorescence signal (negative control) in susceptible reaction potato Irys when primary antibodies were omitted. Bar 50 µm. Ep—epidermis, Me—mesophyll, St—stomata, VB—vascular bundle.

In the present research, we analyzed the localization of RbohD in two types of PVY^NTN^-potato interactions: Susceptible and resistant. The resistant interaction consisted of two hypersensitive type reactions, one with high resistance level to PVY^NTN^ (cultivar Sárpo Mira) and the other with a low resistance to PVY^NTN^ (cultivar Rywal). The susceptible interaction consisted of the potato cultivar Irys. We studied the RbohD detection at two time points for all interactions, before symptoms appeared and when the symptoms were fully developed. The immunofluorescence localization of RbohD in the resistant as well as in the susceptible potato revealed that the RbohD protein was highly induced by PVY^NTN^ inoculation (Figure 1A,B, Figure 2A,B and Figure 3A–C) when compared to mock-inoculated potato tissues (Figure 1C, Figure 2C and Figure 3D), and when primary antibodies were omitted (Figure 1D, Figure 2D and Figure 3E).

The quantification using the CTCF analysis for RbohD localization clearly indicated that the most active and statistically significant antigen signal induction was detected in the highly resistant potato Sárpo Mira (Figure 1E). Unexpectedly, the fluorescence signal induction in the hypersensitive potato Rywal (with lower resistance than Sárpo Mira) was less intense than the signal observed in the compatible PVY^NTN^—Irys interaction. In all types of interactions, we observed a decrease of green fluorescence signal detection when local symptoms developed with a hypersensitive response and systemic necrosis in susceptible reactions. Interestingly, in the two potatoes with lower and high resistant levels, the decrease in intensity was much faster in Rywal than in Sárpo Mira. Moreover, in the compatible interactions, there was a statistically significant stepwise decrease in the RbohD antigen signal. Our findings revealed that at day 1, after PVY^NTN^-inoculation, green fluorescence signal indicating RbohD deposition was observed in the xylem with external and internal phloem as well as in parenchyma cells of the Sárpo Mira (Figure 1A), whereas, in Rywal, this was observed mainly in the phloem (Figure 2A). There was a clear strong green fluorescence RbohD signal in cell walls of all the tissues mentioned above before the hypersensitive symptoms appeared. The RbohD signal was found mainly in the vascular bundles and in the epidermis of Sárpo Mira seven days after inoculation (Figure 1B). The RbohD signal was observed in the xylem tissue and in mesophyll cells of the Sárpo Mira undergoing necrosis three days after PVY ^NTN^-inoculation (Figure 2B). A weak fluorescence signal was observed in the vascular bundles of mock-inoculated resistant and susceptible potatoes plants (Figure 1C, Figure 2C and Figure 3D). In contrast, a lack of RbohD signal was observed in the control of leaf tissues, where primary antibodies were omitted or replaced with normal serum (Figure 1D, Figure 2D and Figure 3E). In the compatible PVY^NTN^–Irys interaction, RbohD signal was found predominantly in the vascular bundles, but also found in the epidermis, especially in stomata seven days after virus inoculation (before necrotic symptoms developed) (Figure 3A,B). When symptoms were fully developed 21 days after inoculation, localization was strictly associated with cells undergoing necrosis, such as single mesophyll cells, vascular tissues, or epidermis (Figure 3C).

### 2.2. Ultrastructural Localization of RbohD Protein in Compatible and Incompatible Interactions

**Figure 4:**
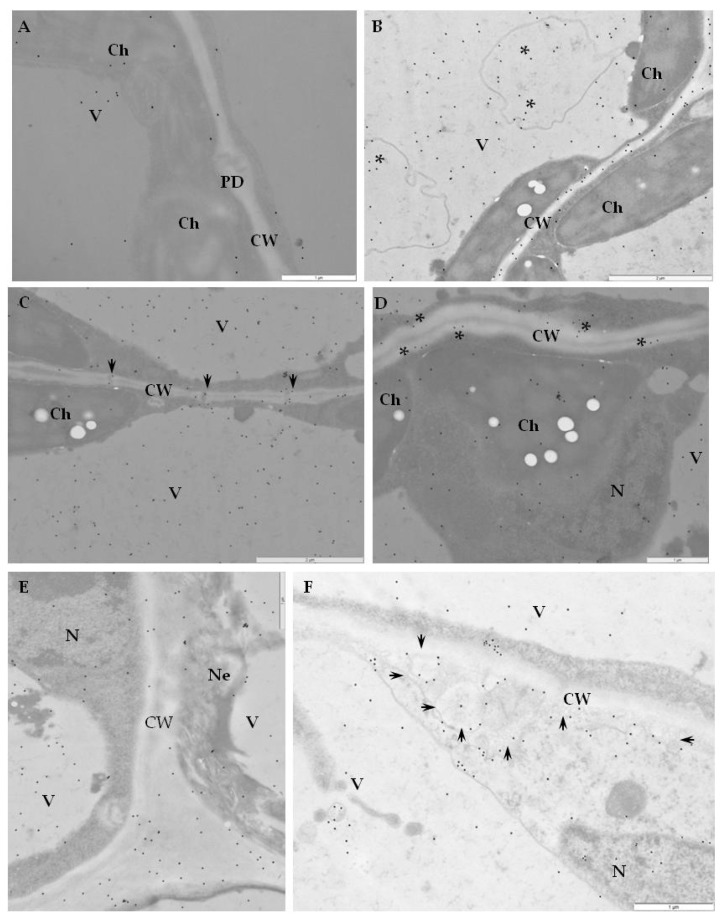
Immunogold labeling of RbohD protein in an incompatible interaction of potato Sárpo Mira plants with PVY ^NTN^. (**A**) Gold particles indicating the presence of RbohD in chloroplast and vacuole in parenchyma cell of mock-inoculated potato Sárpo Mira plant. Bar 1 µm. (**B**) Gold particles indicating the presence of RbohD in the cell wall, vacuoles, and membranous structures (*) 1 day after PVY^NTN^-inoculation. Bar 2 µm. (**C**) RbohD gold deposition in plasmodesmata in the cell wall (arrows) and in the vacuole 1 day after PVY^NTN^ inoculation. Bar 2 µm. (**D**) RbohD localization along cell wall and in vacuole 7 days after inoculation at the early step of necrosis. Localization in chloroplast and cytoplasm. Bar 1 µm. (**E**) RbohD deposition in necrotic epidermis cells 7 days after virus inoculation. Notice RbohD deposition in the cell wall, vacuole, and nucleus. Bar 1 µm. (**F**) Deposition RbohD in paramular bodies along cell wall (arrows), vacuole and nucleus 7 days after virus inoculation. Bar 1 µm. Ch—chloroplast, CW—cell wall, N—nucleus, Pd—plasmodesmata, V—vacuole, Ne—necrosis.

Immunogold labeling revealed that RbohD epitopes were mainly detected in the vesicular, membranous structures, cell walls, and plasmodesmata in resistant Sárpo Mira plants on day 1, but also 7 days after PVY ^NTN^-inoculation when hypersensitive symptoms appeared (Figure 4A–F, Table 1).

The quantification using RLI by immunogold of RbohD epitopes resulted in a statistically significant increase in gold particle deposition, especially in cell walls and vesicles, whereas a decreased deposition in vacuoles in the Sárpo Mira inoculated with PVY^NTN^ when compared to mock-inoculated plants was shown (Figure 4A).

**Figure 5:**
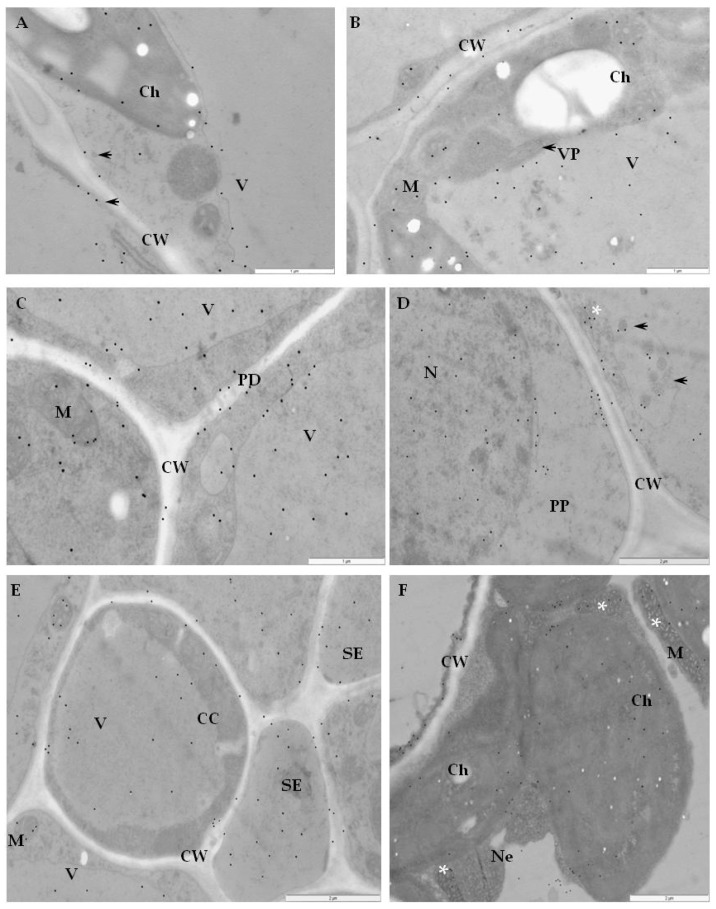
Immunogold labeling of RbohD protein in an incompatible interaction of potato Rywal plants with PVY ^NTN^. (**A**) RbohD antigen gold particles deposition in chloroplast and vacuole of the mesophyll cell. Low deposition of gold particles along the cell wall (arrow) in a mock-inoculated potato Rywal plant. Bar 1 µm. (**B**) RbohD located in chloroplast and mitochondria 1 day after PVY ^NTN^-inoculation. The deposition in the vacuole and cell wall of the mesophyll cell altogether with virus particles in the cytoplasm. Bar 1 µm. (**C**) RbohD antigen gold particles in the mitochondria and vacuole in phloem parenchyma cells 1 day after PVY ^NTN^-inoculation. Gold particles present in vesicles and the cell wall around the plasmodesmata. Bar 1 µm. (**D**) Gold deposition in the nucleus, multivesicular bodies (arrows), phloem cells, and Golgi apparatus (*) 1 dai. Bar 2 µm (**E**) RbohD deposition in the sieve element, and companion cell 3 days after virus inoculation. Gold particles in the vacuole, mitochondria, and along the cell wall. Bar 2 µm. (**F**) RbohD detection in necrotizing phloem cells 3 days after PVY ^NTN^-inoculation. Gold particles mainly in the chloroplast with the ultrastructural changes cell wall, and mitochondria (*). Bar 2 µm. CC—companion cell, Ch—chloroplast, CW—cell wall, M—mitochondria, N—nucleus, Ne—necrosis, PP—phloem parenchyma, SE—sieve element, V—vacuole, VP—virus particles, vs—vesicles.

**Figure 6:**
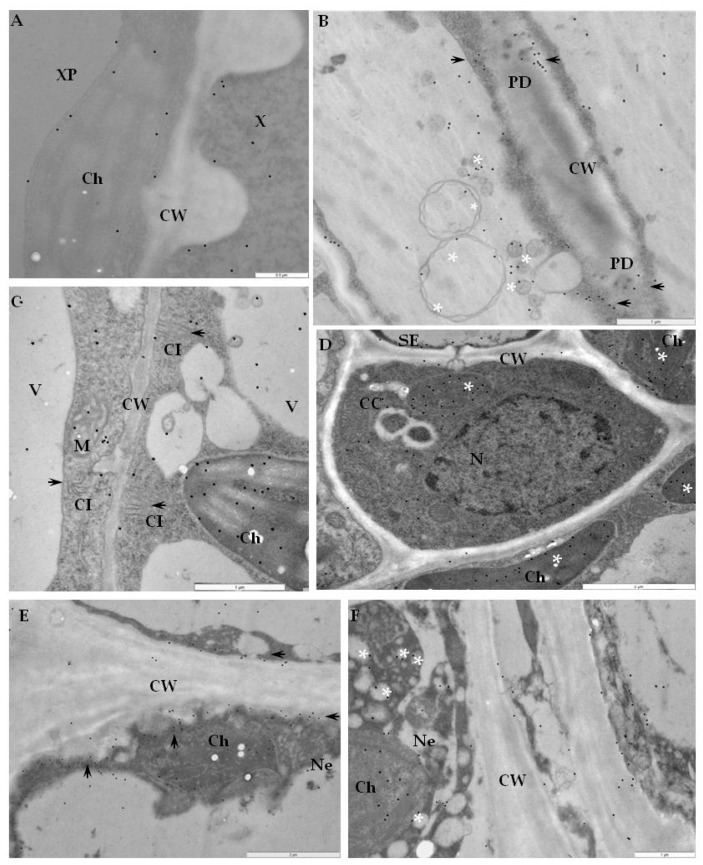
Immunogold labeling of RbohD protein in a compatible interaction of potato Irys plants with PVY^NTN^. (**A**) Gold particles indicating the presence of RbohD in the xylem tracheary element. Deposition of gold particles in chloroplast and cytoplasm of xylem parenchyma cell in mock-inoculated potato Irys plant. Bar 0.5 µm. (**B**) RbohD localization in cell wall near plasmodesmata (arrows), along with vesicular structures (*) and in phloem cells 7 days after PVY^NTN^-inoculation. Bar 1 µm. (**C**) RbohD localization in chloroplast, mitochondria, and vacuole in a mesophyll cell 7 days after PVY ^NTN^-inoculation. Gold particles present in the cytoplasm near virus cytoplasmic inclusions. Bar 1 µm. (**D**) Gold particles indicating the presence of RbohD in phloem cells at the early stage of necrosis, 21 days after PVY^NTN^-inoculation. Gold particles in chloroplast and cytoplasm. Bar 2 µm. (**E**) Gold deposition along cell wall (arrows) of necrotic epidermis cell 21 days after PVY ^NTN^-inoculation. RbohD located in the necrotized cell, chloroplast, and vacuole. Bar 2 µm. (**F**) Gold particles indicating RbohD in necrotic mesophyll cells. Deposition in chloroplast and vesicles (*) 21 days after PVY ^NTN^-inoculation. Bar 1 µm. CC—companion cell, Ch—chloroplast, CI—virus cytoplasmic inclusion, CW—cell wall, M—mitochondria, Ne—necrosis, V—vacuole, X—xylem tracheary element, XP—xylem parenchyma.

Additionally, the strongest deposition of RbohD in cell walls and membranous paramular bodies were noticed 7 days after PVY^NTN^-inoculation, when the hypersensitive response appeared (Figure 4E,F). Moreover, these depositions were usually associated with necrosis (Figure 4E). These findings suggest that RbohD was activated as a result of virus inoculation and that the distribution and deposition changed compared to healthy plants. Interestingly, the gold in the PVY^NTN^–Rywal interaction was usually associated with plant cell organelles 3 days after inoculation (Figure 5A–F, Table 2). Quantification by immunogold of RbohD epitopes resulted in a statistically significant amount of gold particles in the vacuole, Golgi apparatus, and nucleus (Figure 5B–D). The analyzed data indicate that the deposition of RbohD in chloroplasts significantly increased 3 days after inoculation, whereas in the vacuole, it decreased after the hypersensitive response appeared in potato Rywal. Furthermore, the cell wall deposition of RbohD increased in potato Rywal 1 day after PVY^NTN^-inoculation (Figure 5B,C) when compared to mock-inoculated plants (Figure 5A) but decreased 3 days after inoculation (Figure 5E,F). Additionally, both mock-inoculated resistant plants, independent of the resistance level, revealed RbohD deposition in the vacuole, Golgi apparatus with vesicles, and chloroplasts (Figure 4A and Figure 5A). In conclusion, different levels of RbohD deposition and distribution patterns in potato cells were dependent not only on the host resistance level to PVY^NTN^ but also on the level of necrosis. Moreover, the analyses of deposition of RbohD in compatible PVY^NTN^—Irys indicated that the RbohD protein was located predominantly in chloroplasts, Golgi apparatus, and mitochondrion 7 days after inoculation, when symptoms had not yet developed (Table 3, Figure 6B,C). When the symptoms of systemic necrosis in the susceptible potato Irys were visible, RbohD deposition in chloroplasts and cytoplasm occurred, however, it was strictly associated with cells undergoing necrosis, which included companion cells (Figure 6D), the epidermis (Figure 6E), and mesophyll (Figure 6F). These results indicated that even when the susceptible plant did not react with a rapid localized response, a significant distribution of RbohD was noticed.

### 2.3. Detection of H_2_O_2_ and Virus Particles Location in Susceptible and Hypersensitive Reaction to PVY^NTN^

**Figure 7:**
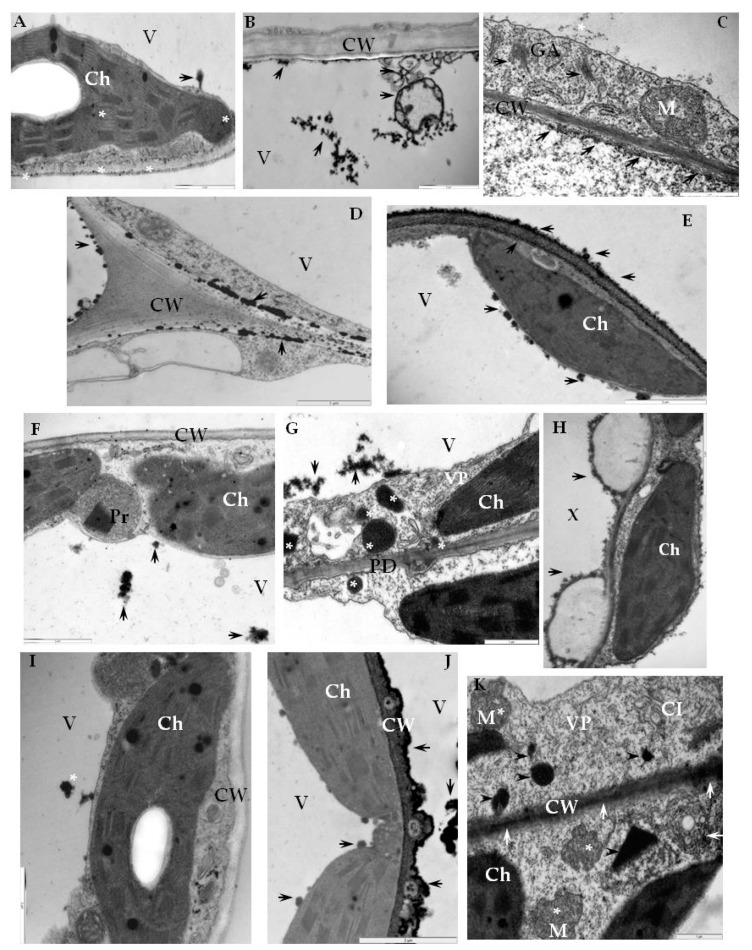
Ultrastructural detection of H_2_O_2_ in an incompatible (B–E, G–H) and compatible (J,K) interactions of potato with PVY^NTN^. (**A**) H_2_O_2_ deposits (*) along the cell wall in the vacuole (arrow) and of mock-inoculated Sárpo Mira leaf (control). Bar 2 µm. (**B**) H_2_O_2_ deposits (arrows) in stained membranous, vesicular structures inside the vacuole 1 day after PVY^NTN^-inoculation of potato Sárpo Mira. Bar 1 µm. (**C**) Electron dense deposits of cerium perhydroxide precipitate along with the Golgi apparatus, along the cell wall and in the vacuole 1 day after PVY^NTN^-inoculation in mesophyll cell potato Sárpo Mira. Bar 1 µm. (**D**) H_2_O_2_ deposits (arrows) between the cell wall and plasmalemma and along the cell wall 7 days after PVY inoculation of potato Sárpo Mira mesophyll cell. Bar 1 µm. (**E**) H_2_O_2_ along cell wall and in vacuole along tonoplast (arrows) of a mesophyll cell 7 days after PVY^NTN^ inoculation of potato Sárpo Mira mesophyll cell. Bar 2 µm. (**F**) H_2_O_2_ in the vacuole in mock-inoculated potato Rywal plant. Bar 1 µm. (**G**) Vacuole, cytoplasm, peroxisomes, and cell wall near the plasmodesmata stained with cerium perhydroxide (*) in mesophyll cells 1 day after PVY^NTN^-inoculation potato Rywal. Virus particles in the cytoplasm. Bar 1 µm. (**H**) H_2_O_2_ deposition along the cell wall (*) in xylem tracheary elements 3 days after PVY^NTN^ inoculation of potato Rywal. Bar 2 µm. (**I**) Weak deposition of H_2_O_2_ (*) in the vacuole of mesophyll cell of mock-inoculated potato Irys plant. Bar 2 µm. (**J**) H_2_O_2_ (arrows) along the cell wall between necrotized and non-necrotized cells. H_2_O_2_ also in the vacuole (*) 7 days after virus inoculation of potato Irys mesophyll cell. Bar 2 µm. (**K**) H_2_O_2_ precipitates (*) in non-necrotized cells in changed mitochondria, along membranes of multivesicular bodies as well as cell wall (arrows). H_2_O_2_ inside distorted peroxisomes and in the cytoplasm (arrowhead) with PVY particles and cytoplasmic inclusions 21 days after PVY^NTN^-inoculation of Irys mesophyll cells. Bar 1 µm. CI—virus cytoplasmic inclusion, Ch—chloroplast, CW—cell wall, GA—Golgi apparatus, M—mitochondria, Pr—peroxisome, PD—plasmodesmata, V—vacuole, VP—virus particles, X—xylem tracheary elements, XP—xylem parenchyma.

**Figure 8:**
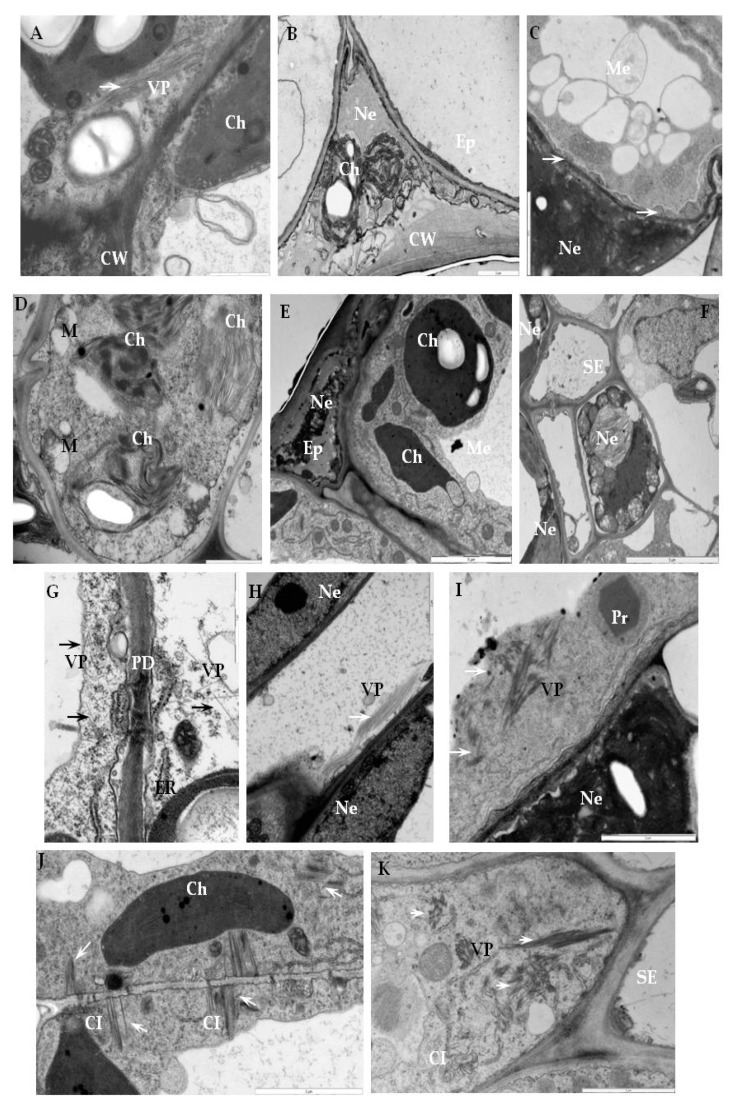
Ultrastructural location of virus particles in an incompatible interaction of potato Sárpo Mira (A–D) and Rywal plants (E–H) and compatible interaction of potato Irys plants (I–K) with PVY ^NTN^. (**A**) PVY^NTN^ particles (arrow) in mesophyll cells of the inoculated area 1 day after PVY^NTN^-inoculation of potato Sárpo Mira. Bar 1 µm. (**B**) Necrosis in epidermis 7 days after PVY^NTN^-inoculation; notice the lack of virus particles. Destruction of chloroplasts in stomata and changed cell wall. Bar 2 µm. (**C**) Necrosis in mesophyll 7 days after PVY^NTN^-inoculation of potato Sárpo Mira. Notice the lack of virus particles and changed cell wall near the plasmodesmata areas (arrows). Bar 2 µm. (**D**) Changed ultrastructure of chloroplasts and mitochondria 7 days after PVY^NTN^-inoculation outside of the inoculation area of potato Sárpo Mira. Notice the lack of virus particles. Bar 1 µm. (**E**) Necrotization of epidermis 1 day after PVY^NTN^-inoculation potato Rywal. Bar 5 µm. (**F**) Necrotic cells in phloem tissue 3 days after PVY ^NTN^-inoculation of potato Rywal. Bar 5 µm. (**G**) PVY^NTN^ particles (arrows) 3 days after PVY ^NTN^-inoculation in mesophyll cell next to plasmodesmata outside the inoculation area of potato Rywal. Bar 1 µm. (**H**) Virus particles in phloem cell outside the inoculation area of potato Rywal 3 days after PVY ^NTN^-inoculation. Bar 2 µm. (**I**) Virus particles (arrows) in potato Irys mesophyll cell (inoculated area) 7 days after inoculation. Bar 2 µm. (**J**) Virus cytoplasmic inclusions associated with plasmodesmata (arrows) in Irys mesophyll cells (outside the inoculation area) 21 days after inoculation. Bar 2 µm. (**K**) Virus particles (arrows) and cytoplasmic inclusions in Irys phloem cells (outside the inoculation area) 21 days after inoculation. Bar 2 µm. Ch—chloroplast, CI—virus cytoplasmic inclusion, CW—cell wall, Ep—epidermis, ER–endoplasmic reticulum, M—mitochondria, Me—mesophyll, Ne—necrosis, Pr—peroxisome, PD—plasmodesmata, SE—sieve element, VP—virus particles.

In plant cells, the generation of ROS like H_2_O_2_ requires the activity of NADPH oxidases, including RbohD. Moreover, H_2_O_2_ is one of the most abundant and stable ROS-generated molecules inside cells. Therefore, in this part of the study, we determined the H_2_O_2_ localization in the three types of PVY^NTN^-potato interactions described above, including the association with RbohD. In order to localize H_2_O_2_ in tissues of PVY^NTN^-resistant and susceptible potato, a method which allows the detection of cerium IV perhydroxide precipitates was used [28,29]. The localization of electron-dense deposits, indicating H_2_O_2_ presence, confirmed the main cell areas of RbohD detection in the three different types of PVY^NTN^-potato interaction (Figure 7A–K). The cerium IV perhydroxide precipitates were concentrated along with multivesicular bodies and inside vacuoles starting from 1 day after PVY^NTN^-inoculation of Sárpo Mira (Figure 7B). Analysis of the micrographs indicated that the production of H_2_O_2_ was induced when compared to mock-inoculated plants, independently of the interaction type (Figure 7). Moreover, H_2_O_2_ was also deposited along the cell wall and membranous structures such as the Golgi apparatus (Figure 7C), whereas the most extensive deposition, was noticed between the cell wall and plasmalemma or along the cell wall 7 days after PVY^NTN^-inoculation (Figure 7D,E) when hypersensitive symptoms were visible in the Sárpo Mira. The deposition in healthy, mock-inoculated potato plants (resistant and susceptible) was associated with chloroplasts and vacuoles (Figure 7A,F,I). As our previous investigations [29] in the PVY^NTN^-potato Rywal interactions, the H_2_O_2_ was clearly induced when compared with healthy plants. Vacuole, cytoplasm, and peroxisomes, as well as cell wall and plasmodesmata, were places where H_2_O_2_ deposited (Figure 7F–H). The most intense depositions were observed in areas where PVY particles were present, such as the mesophyll or xylem cells (Figure 7G,H). Unexpectedly, active H_2_O_2_ deposition was found in the late stages of infection in the susceptible potato Irys (Figure 7J,K). Before systemic symptoms developed, H_2_O_2_ was located usually along the cell wall, especially in cells undergoing necrosis, whereas, in non-necrotic cells, localization of H_2_O_2_ was observed in the mitochondria, along membranes of multivesicular bodies as well as the cell wall (Figure 7J,K). Moreover, hydrogen peroxide was observed inside the chloroplast, peroxisomes, and in the cytoplasm where PVY particles and cytoplasmic inclusions were present (Figure 7K). In relation to H_2_O_2_ localization, virus particle locations were analyzed in the three types of PVY^NTN^-potato interaction. In the Sárpo Mira–PVY^NTN^ interaction, virus particles were observed rarely in the inoculation area 1 day after inoculation (Figure 8A). Whereas, 7 days after inoculation, PVY particles were not observed when necrosis of the epidermis or mesophyll cells were developed, accompanied with cell wall rearrangements and ultrastructural alteration of plant cell organelle (Figure 8B–D). One day after inoculation of the potato Rywal, single epidermis or mesophyll cell necrosis started and PVY^NTN^ particles were presented in all tissues in the inoculation area (Figure 8E). In contrast to the potato Sárpo Mira plants, when the hypersensitive symptoms were visible, virus particles were also detected outside the inoculation area in mesophyll cells, near the plasmodesmata or in vascular tissues (Figure 8G,H). One day or 3 days after potato Rywal inoculation, when the symptoms were visible, the virus cytoplasmic inclusion were not observed. Moreover, the necrotization process in the mesophyll cells and phloem and xylem tissue were noticed (Figure 8E,F,H). In the susceptible potato Irys, PVY^NTN^ particles and inclusions were observed in the inoculation area and outside the inoculation in all tissue types (Figure 8I–K). The necrotization development was accompanied by the presence of virus particles in the mesophyll cell cytoplasm, also with virus inclusions associated with plasmodesmata. Furthermore, virus particles were noticed in both vascular tissues.

These findings clearly indicate the variable effects of different types of PVY^NTN^ interactions. They occurred not only due to a rapid oxidative burst but also as an effect of a late infection associated with the systemic necrosis reaction in the susceptible potato plants.

## 3. Discussion

Plants have evolved to survive through actively accommodating to rapid changes in the environment. The generation of ROS has been suggested as one strategy to react to biotic and abiotic stress factors [30]. Moreover, ROS transduce local as well as systemic signaling for adaptation or tolerance to these stresses. The successful infection process by a plant virus depends on its ability to overcome the host plants barriers. The roles of ROS and oxidative stress in plant–virus interactions were postulated by Doke and Ohashi [12], highlighting the association of rapid oxidative burst with the host resistance reaction. Most of the research about the involvement of the ROS in plant–virus interactions, especially interactions with members of the genus *Potyvirus,* has been concentrated on the oxidative stress enzyme activity and regulation. It has been reported, that ZYMV infection influenced proline oxidase, superoxide dismutase, as well as catalase activities in *Cucumis* and *Cucurbita* species [27,31]. The antioxidative metabolism has also been characterized in Plum pox virus (PPV, *Potyvirus*)—*Prunus* sp. interactions, where in different host resistance levels, different activities of catalase and superoxide dismutase were noticed [32]. Additionally, the importance of GSH in symptoms development was also confirmed. Moreover, in plants systemically infected with Sunflower chlorotic mottle virus, superoxide dismutase (SOD) and CAT activity increased before the symptoms appeared [33]. It has been postulated that because of PVY ^NTN^-susceptible potato Igor interactions, high expression of genes related to the antioxidant metabolism like ascorbate peroxidase (APX), glutathione peroxidase (GPX), or glutathione reductase (GR) with glutathione-S-transferase (GST) occurs [34]. Interestingly, when another susceptible cultivar Nandine was used and a mild strain of PVY, the reaction was the opposite and authors suggested a host-dependent different antioxidant regulation.

A two branched innate immune system has been proposed by Jones and Dangl [35] as the response of plants to pathogen infection. Firstly, the pathogen or microbe associated molecular pattern (PAMP’s or MAMP’s) in which PRR (plant pattern recognition receptors) form basal immunity, which is known as PAMP (or MAMP) triggered immune response (PTI). PAMPs included conserved molecules such as bacterial flagellin, polysaccharides, fungal chitin, or glucan [36]. Plants may disrupt host or even non-host invaders through PTI [37]. The second basic plant response involves intercellular receptors of pathogen virulence, named effectors (effector-triggered immunity-ETI) or resistance gene (R)-mediated effectors- triggered immunity [38,39]. Plant viruses do not code for PAMPs or ETIs and the antiviral immune response is triggered by resistance proteins which are not classified as ETI yet. The generation of ROS during an oxidative burst is dependent on respiratory burst oxidase homologs and it is strictly associated with the pathogen recognition process leading to the perception of MAMPs/PAMPs and during the hypersensitive response [40]. The role of RBOHs in the plant–viral pathogen interaction has been investigated in different plant species by an antisense approach or knockout plants. In *A. thaliana*, RbohD and RbohF are responsible for the ROS burst as an effect of MAMPs [23]. Moreover, RbohB and RbohA are the main ROS producer in *Phytophtora infestans* [26] and *Botrytis cinerea* infections [25]. In *N. bethamiana*, RbohD is responsible for the oxygen burst induced by cryptogein from oomycetes [24]. In recent years, there have been many reports on the role of respiratory burst oxidases in plant–pathogen interactions, although our knowledge about the relationship between host–virus interaction and RBOHs is still poor. The investigations of Doke and Ohashi [12] revealed that NADPH-calcium dependent generation of O_2_^−^ take place in membrane-rich fractions of TMV-infected tobacco plants with the N-resistance gene, which suggested a strong oxidative burst in the virus-resistance reaction. They also indicated that O_2_^−^ was produced by membrane bound NADPH-oxidase. It has been reported that the NADPH-oxidase reaction recruits a secondary structure of TMV coat protein in the TMV-triggered early phase of ROS burst [41]. Additionally, Moeder et al. [42] postulated the role of NADPH oxidase-dependent superoxide production in N-gene mediated resistance to TMV. It has been reported that HR-localized programmed cell death is associated with pathogen restriction in the inoculated areas, leading to necrotic symptoms. Therefore, in the present investigation, we analyzed the localization of the key and major source of reactive oxygen production—NADPH oxidase RbohD protein in potato with different resistance levels to PVY^NTN^. Moreover, we also demonstrated, at the ultrastructural level, that RbohD is differentially expressed in compatible as well as incompatible PVY^NTN^—potato interactions. Our studies indicated that PVY^NTN^ inoculation strongly induced RbohD, not only in resistance but also in susceptible potato plants. NADPH oxidase RbohD is considered as the main source of the oxidative burst in the apoplast of *A. thaliana–Alternaria* patho-systems and in the hypersensitive potato Sárpo Mira with a high level of resistance [43]. In our investigations, we noticed a high induction of RbohD associated with the cell wall and forming paramular bodies. Moreover, our previous investigations revealed a cell wall thickening of Sárpo Mira potato, which was accomplished by the formation of vesicular structures [44]. Therefore, the high deposit of RbohD in the apoplast area observed in this study supports the findings that RbohD could mediate the lignification process [45]. If RbohD mediates the cell wall reinforcement in resistant host plants during the plant virus–host interaction, it can also restrict the pathogen or even block it, especially in host plants with high resistance levels like Sárpo Mira. One day after inoculation, of the hypersensitive cultivar Rywal (which reacted with a high resistance level, but lower than Sárpo Mira) the RbohD deposition in the cell wall was not as high as in the case of Sárpo Mira. Furthermore, when the hypersensitive reaction symptoms on the Rywal leaves were visible (3 dai), the RbohD cell wall deposition was not statistically significant. Additionally, as we previously reported [46], PVY ^NTN^ was able to move from the inoculated to the upper leaves, which suggest that it was not completely blocked as typically happens in a hypersensitive reaction to a pathogenic infection. It is possible that the more intense accumulation of RbohD in the cell wall and other apoplast areas, the more successful the control of PVY. It has been reported that in the case of biotrophic pathogens–host interactions, such as in the case of *Arabidopsis–Golorinoomycetes cichoracearum* [47] or *Hordeum vulagare–Blumeria graminis* [48], there is an absence of expression of NADPH oxidase genes leading to host susceptibility. These findings indicate that NADPH oxidases-derived ROS had a role in limiting pathogen and host cell death initiation [49], and as a result promoted cell wall reinforcement in the infected plant cells. As we highlighted earlier, the role of ROS and respiratory burst oxidases homologs in plant virus interactions have not been fully studied. Interestingly, it has recently been reported that Red clover necrotic mosaic virus (RCNMV, *Dianthovirus*) hijacks the ROS-generating machinery of the host [50]. Moreover, RCNMV replication was sensitive to an O_2_^−^ scavenger but insensitive to H_2_O_2_. Furthermore, RCNMV triggered intercellular ROS burst but silenced the RBOH genes inhibiting RCNMV infection in the plants. The authors postulated that RbohB in *N. benthamiana* was responsible for the inhibition of pathogen infection in plant innate immunity [50].

In the present study, independently of the resistance level, RbohD was mainly found in the Golgi apparatus and vesicular structures. After the virus inoculation, the RbohD deposition in the Golgi apparatus was always statistically significant in the analyzed tissues. Our findings were compatible with the data of Sagi and Fluhr [51] and Takada et al. [52], who postulated RbohD localization in clusters near the plasma membrane. However, the most common place for RbohD deposition was the Golgi cisternae [14]. Our findings underlined the active participation of the host plant cell organelles in RbohD localization after PVY^NTN^-inoculation. In our studies, in both the hypersensitive reaction types, the distribution of RbohD as well as deposition of H_2_O_2_ was different. As mentioned before in the high resistant to PVY^NTN^ potato Sárpo Mira plants, the localization of RbohD was concentrated around the apoplast cell wall, paramular bodies, membranous structures, Golgi apparatus, and vesicles. Whereas, in the low resistant potato Rywal we noticed that especially 1 day after inoculation, that the PVY ^NTN^ changed the distribution of RbohD from the vacuole, Golgi, and within vesicles to other organelles, especially the mitochondria, nucleus, and chloroplasts. Moreover, when hypersensitive symptoms developed, three days after inoculation, a high deposition of RbohD was mainly found in the chloroplasts and mitochondria. The RbohD cell wall localization was also observed, but with less intensity than in the resistant cultivar Sárpo Mira. The NADPH oxidase activity associated with the generation of H_2_O_2_ in PVY ^NTN^-Rywal that occurred within the plant cell organelles seems to be the result of ROS-related biochemical processes. Moreover, plant viruses, in contrast to other pathogens, are not only closely associated with the plant cell cytoplasm, but also with cellular organelles such as chloroplast, nucleus, mitochondria, and membranes [5,46]. In the present study, we noticed a high level of accumulation of RbohD and H_2_O_2_ in the chloroplast between 1 and 3 days after PVY^NTN^-inoculation, when the hypersensitive necrotic symptoms developed. Pathogen stress, like viruses, may further decrease the CO_2_ fixation of photosynthesis, leading to an enhanced ROS accumulation in the chloroplast [53,54,55]. Plant defense related oxidative burst occurs in chloroplastic PSI and PS II [56]. It has been shown that infections by tobamoviruses facilitate the inhibition of PSII electron transport by disturbing the oxygen-evolving complex (OEC) [57]. Moreover, Rahoutei et al. [58] reported that two PSII OEC proteins, PsbP and PsbQ, were lower in plants infected with Pepper mild mottle virus (PMMoV) when compared to healthy plants. Furthermore, the virus infection affected the oxygen concentration of the thylakoid membranes. According to Balasubramaniam et al. [59], PsbP interacts with the capsid protein of the Alfalfa mosaic virus (AMV, *Alfamovirus*). In high concentrations, PsbP may inhibit virus replication and this could result in specific chloroplast-related resistance to AMV and thus ROS generation.

The induction of RbohD in the susceptible PVY^NTN^-potato Irys interaction provides evidence for the involvement of RbohD in pathogen stress. RbohD was mainly localized in the vascular tissue and tissues developing necrosis. Our findings are similar to the results of analyses of differential gene expression of *Arabidopis* plants infected with Tobacco etch virus (TEV, *Potyvirus*) reported by Hillung et al. [60]. TEV isolates infecting the *Arabidopis* ecotype St-0 showed symptoms ranging from moderate to severe. Furthermore, most of the up-regulated gene categories resulting from the transcriptome analysis were largely implicated in respiratory burst activities [60]. In our study, in the susceptible PVY^NTN^-potato Irys interaction, the RbohD protein was found at an early stage of infection (7days after PVY^NTN^-inoculation). The RbohD protein was mainly associated with the chloroplast, mitochondria, and vacuole. In the cell wall, the deposition of RbohD was very low, in mock-inoculated potato Irys at both time points of the compatible interaction (7 and 21 days after PVY^NTN^-inoculation). During the systemic infection, the deposition of RbohD was in the plasmodesmata. At the late step of infection, (21 days after PVY^NTN^-inoculation) when the symptoms were fully developed, RbohD antigen deposition was in the chloroplast and cytoplasm at statistically significant levels. Moreover, it should be emphasized that the deposition was strictly associated with necrotic areas in the epidermis, mesophyll, and phloem cells. These findings suggest that symptoms development is associated with the compatible PVY^NTN^-potato Irys interaction and with a different distribution of RbohD. It appears that during compatible virus interaction, senescence symptoms are induced in plants and as the senescence progresses, chloroplasts are the most affected cell organelles. This organelle could be a source of oxidative stress during the progression of the viral infection. Similarly, the correlation of high ROS levels with susceptibility has been reported by Schafer et al. [61] in barley infected with a hemibiotrophic fungal pathogen. Diaz-Vivancos et al. [7,8] reported an enhanced accumulation of H_2_O_2_ in *Prunus* sp. systemically infected with PPV. The strong systemic burst of H_2_O_2_ in these plants were explained by a display of chlorosis and failure by the host to elicit resistance in the systemically infected vascular tissue.

In summary, in the susceptible PVY ^NTN^-potato Irys interaction, RbohD induction, and active distribution were associated with systemic symptoms development, leading to systemic necrosis. Additionally, RbohD deposition was high in necrotizing tissues or cells. RbohD has been found to mediate long distance systemic signaling [16]. The localization of RbohD together with H_2_O_2_ deposition in plasmodesmata was physical evidence of the oxidative burst transport. Cui and Lee [62] demonstrated that exogenous ROS decrease plasmodesmatal permeability in *A. thaliana* foliar tissues. It is possible that cytosolic H_2_O_2_ generated from RbohD activation could diffuse via plasmodesmata. Another possibility is that ROS may travel through the phloem and xylem which would enable the long distance movement [19,63]. Therefore, the ROS signal could be from cell-to-cell through vesicles containing H_2_O_2_.

## 4. Material and Methods

### 4.1. Plants and Virus

*Solanum tuberosum* plants of 3 cultivars with different resistance levels (in a 1–9 scale): -Irys (PVY^NTN^ resistance score 5.5), -Rywal (PVY^NTN^ resistance score 8) and -Sárpo Mira (resistance score 9) [64] were acquired from the Plant Breeding and Acclimatization Institute, Bonin Research Center (IHAR-PIB), Bonin, Poland. Plants were grown in a greenhouse and mechanically inoculated at the five-leaf stage with the NTN strain of PVY as previously reported [44,65]. Of the potato cultivars Irys, Rywal, and Sárpo Mira, 25 plants were inoculated in every experiment. This experiment was repeated 3 times. Sárpo Mira developed a hypersensitive necrotic response visible 7 days after inoculation, whereas Rywal at 3 days after inoculation. In the Sárpo Mira, it is known that the reaction is conferred by the *Ny-Smira* gene located on the long arm of the potato IX chromosome [66], whereas in Rywal by the *Ny-1* gene located on the short arm of chromosome IX. Cultivar Irys developed systemic necrosis visible 10 days after inoculation. Leaves from PVY^NTN^-infected plants (3 leaves from every plant) were collected at two different time intervals depending on the type of the reaction. In the case of susceptible potatoes (Irys), the leaves were collected 7 and 21 days after -PVY^NTN^ inoculation, whereas in the case of the resistant potatoes Sárpo Mira, leaves were collected 1 and 7 dai, Rywal leaves were collected 1 and 3 dai. Different starting points for collecting the plant material were chosen because of differences in the course of viral infection on either cultivar. Healthy leaves of the 3 cultivars (used as controls) were mock-inoculated with 0.01 M phosphate buffer 7 dai. The ELISA test was used to confirm that the three potato cultivars were free of PVY [67].

### 4.2. Immunofluorescence Localization of RbohD and the Assessment of the Quantitative Fluorescence Signal

For immunofluorescence localization and quantification of RbohD fluorescence signal, 3 leaves from each of the 25 plants at different time intervals depending on the type of the reaction, were cut for sections. This stage was repeated 3 times for each one of the 3 cultivars.

From the PVY^NTN^ and mock-inoculated potato plants (at described time intervals), 2 mm^2^ sections of leaves were fixed and embedded in butyl-methyl-methacrylate (BMM) resin according to a procedure described previously [65], with modifications [68]. Acetone was used to remove the BMM from 2 μm sections and placed in silane slides (Thermo-Fisher Scientific, Warsaw, Poland). Immunofluorescence procedure/analysis was carried out as described by Otulak et al. [44]. For the localization of RbohD, RbohD-specific IgG from rabbit (primary antibody) was acquired from Agrisera (Vänäs, Sweden), whereas anti-rabbit IgG conjugated with AlexaFluor® 488 (secondary antibody) was provided by Jackson ImmunoResearch Europe Ltd. (Cambridgeshire, UK). The controls consisted of mock-inoculated tissue, detection with a pre-immune serum, and when primary antibodies were omitted. An Olympus AX70 Provis (Olympus Poland, Warsaw, Poland) with a UM61002 filter set and an Olympus UC90 HD camera (Olympus Poland) were used for fluorescence imaging. Images were acquired using the Olympus Cell Sense Standard Software (Olympus, Center Valley, PA, USA, version 1.18). The intensity of the green fluorescent signal from regions of localization of RbohD was further analyzed by use of a quantitative measuring method—corrected total cell fluorescence (CTCF) [69,70] with modifications [68]. For CTCF 25, selected areas of every sample were analyzed. To measure the fluorescent signal levels, we used Image J program (National Institute of Health., Bethesda, MD, USA, version 1.51k). Measurements of the green immunofluorescence signal gained from the Image J program were calculated with CTCF on magnification 20× with 1.00 zoom factor by using the formula:

CTCF = Integrated Density–(Area of selected cell region × Mean fluorescence of background readings)

Estimated CTCF values were then analyzed statistically at selected time intervals for all plants infected with PVY^NTN^ (susceptible and with different level of resistance) by using one-factor analysis of variance (ANOVA). The ANOVA analyses enabled us to determine the values of statistical significance during quantifying the levels of RbohD. Mean values CTCF were calculated at the *p* < 0.05 level of significance using a post-hoc Tukey HSD test in STATISTICA software (StataSoft and TIBCO Software Inc., Palo Alto, CA, USA, version 13.0).

### 4.3. Ultrastructural Virus Particles Location and Hydrogen Peroxide Detection

The hydrogen peroxide was detected in potato leaf tissues mock-inoculated and inoculated with PVY^NTN^ at the time points described in Section 4.1. The method of Bestwick et al. [28] was used with modifications as described in Otulak and Garbaczewska [29]. The tissues were pre-incubated in 5 mM CeCl_3_ in a 50 mM (*w*/*v*) buffer MOPS (pH 7.2) for 1 h, then washed in the same buffer. To the ultrastructural virus particles location analysis, the first step of pre-incubation was omitted. The sections were fixed in 2% (*w*/*v*) paraformaldehyde and 2% (*v*/*v*) glutaraldehyde in 0.05 M cacodylate buffer (pH 7.2–7.4) for 2 h at room temperature [71]. The fixed samples were contrasted and fixed in 2% (*w*/*v*) OsO4 in cacodylate buffer for 2 h at 4 °C. The samples were rinsed with sodium cacodylate and then dehydrated in a series of increasingly ethanol–water dilutions. The material was gradually saturated with resin Epon 812 (Fluka) and polymerized for 24 h at 60 °C. Observations were made as previously described in reference [68].

### 4.4. Ultrastructural Localization of RbohD and Quantitative Immunogold Localization by Direct Estimation of the Relative Labeling Index (RLI)

Potato lea tissues were fixed, embedded, and sectioned for transmission electron microscopy (TEM) as previously reported [44]. Sections (50–60 nm thick) from PVY^NTN^-inoculated or mock-inoculated plants were mounted on Formvar-coated nickel grids and processed as described by Otulak et al. [65]. Grids were rinsed with RbohD specific IgG (primary antibody) sand washed with PBS-Tween 20. Grids with sections were treated for 1 h with 20 nm gold-conjugated (secondary antibody) anti-rabbit IgG (Sigma-Aldrich, Warsaw, Poland), rinsed for 5 min in PBS and then in distilled water. Labeling specificity was checked by incubating grids with material from mock-inoculated plants and omitting the primary antibody in the incubation solution [44]. The grids were counterstained with 1% uranyl acetate for 5 min and washed 5 × 2 min with distilled water. The immunogold-treated sections were examined by transmission electron microscopy [68]. The quantitative assessment of preferential labeling of RbohD to specific structures or organelles was carried out by using relative labeling index (RLI) as described by Mayhew [72] and Otulak et al. [73]. RLI is an approach to test for non-random immunogold labeling of organelles and membranes. The direct estimation method of RLI was used by comparing the number of observed gold particles (G0) within selected compartments with the expected gold particles (Ge) of the appropriate reference structure or organelles in a leaf [72]. For estimation of G0 and Ge, gold particles were scored in 40 of 10 μm2 fields per photo. When there was random labeling, RLI equaled 1, but where there was preferential labeling, RLI was higher than 1. Statistical significance of preferential labeling was assessed by partial Χ2 analysis according to Mayhew [72]. The statistically significant RLI values were >1, with the corresponding partial Χ2 values accounted for a significant proportion (at least 10%) of total Χ2.

## 5. Conclusions

One of the most intensively expressed NADPH oxidase enzymes in pathogen response processes is the respiratory burst oxidase homologue D (RbohD). Additionally, RbohD-dependent reactive oxygen species (ROS) have been reported to participate in plant defense reaction to pathogens and are also associated with the host hypersensitive response. This study demonstrated the RbohD localization and distribution in PVY ^NTN^-potato plants interaction with different resistance levels. Regardless of the type of reaction, RbohD inductions after PVY^NTN^ inoculations were obtained. In the hypersensitive response of potato Sarpo Mira to PVY^NTN^ infection, cell wall reinforcement was followed by virus limitation and RbohD accumulation in the apoplast. This resulted in limiting the virus from the inoculated areas. In contrast, the hypersensitive response of potato Rywal and PVY ^NTN^ resulted in lower RbohD accumulation, accompanied by H_2_O_2_ deposition in the cell wall. However, there was more intense accumulation in the plant cell organelles. There was not enough RbohD accumulation in the apoplast to limit the virus. Interestingly, our findings revealed that in the susceptible interaction PVY^NTN^-potato Irys, the RbohD antigen signal was also significantly obtained. A high deposition of RbohD was associated with the development of necrosis. RbohD was also associated with organelles, particularly the chloroplast; an important plant cell organelle in the context of systemic necrotic lesions. There was a clear deposition of RbohD and H_2_O_2_ in the vascular tissues and plasmodesmata in susceptible interactions. This suggests that RbohD and ROS are involved in oxidative signal systemic transduction. These studies focused on the detection of RbohD and its potential involvement in defense signaling during different types of PVY^NTN^–potato interactions. This is the first report on the effect of PVY^NTN^ on the production and subcellular localization of RbohD in the potato. Further molecular and cellular studies using other virus/host systems are needed to completely elucidate the mechanism and the full spectrum of signal transduction as an effect of plant viral infections.

## Figures and Tables

**Table 1:** Quantification of immunogold labeling of RbohD by Relative Labeling Index (RLI) and Χ^2^ tests in mock-inoculated and PVY^NTN^-inoculated. Sárpo Mira (resistant) potato plants before hypersensitive reaction (HR) symptoms (1 dai) and after HR symptoms developed (7 dai). Significant values (RLI > 1, and % Χ^2^ at least 10%) in bold font and marked with an asterisk.

Sample	Parameters of Immunogold Labeling				
	*G_0_*	*Ge*	*RLI*	*Χ^2^ Value*	*Χ^2^ as %*
Immunogold Localization of RbohD in Potato Sárpo Mira (Resistant):					
**1. Mock-inoculated potato Sárpo Mira plants**					
cell wall	1	1	1.00	0.00	0.00
nucleus	8	6	1.33	0.67	0.55
endoplasmatic reticulum (ER)	2	6	0.33	2.67	2.19
Golgi apparatus	10	2	5.00 *	32.00	26.28 *
chloroplasts	10	2	5.00 *	32.00	26.28 *
mitochondrion	1	5	0.20	3.20	2.63
vacuole	30	9	3.33 *	49.00	40.24 *
cytoplasm	1	4	0.25	2.25	1.85
vesicles	10	3	3.33 *	16.33	13.41 *
Column total				121.78	
**2. 1 day after PVY^NTN^-inoculation potato Sárpo Mira plants**					
cell wall	30	7	4.29 *	75.57	47.72 *
nucleus	8	6	1.33	0.67	0.42
endoplasmic reticulum (ER)	2	6	0.33	2.67	1.68
Golgi apparatus	10	2	5.00 *	32.00	20.21 *
chloroplasts	12	6	2.00	6.00	3.79
mitochondrion	1	5	0.20	3.20	2.02
vacuole	27	9	3.00 *	36.00	22.73 *
cytoplasm	1	4	0.25	2.25	1.42
vesicles	32	7	4.57 *	89.29	56.38 *
Column total				158.35	
**3. 7 days after PVY^NTN^ inoculation potato Sárpo Mira plants**					
cell wall	40	10	4.00 *	90.00	50.62 *
nucleus	18	6	3.00 *	24.00	13.50 *
endoplasmic reticulum (ER)	2	6	0.33	2.67	1.50
Golgi apparatus	12	2	6.00 *	50.00	28.12 *
chloroplasts	24	19	1.26	1.32	0.74
mitochondrion	4	5	0.80	0.20	0.11
vacuole	12	6	2.00	6.00	3.37
cytoplasm	16	10	1.60	3.60	2.02
vesicles	39	10	3.90 *	84.10	47.31 *
Column total				177.78	

**Table 2:** Quantification of immunogold labeling of RbohD by RLI and Χ^2^ tests in mock-inoculated and PVY^NTN^-inoculated Rywal (less resistant) potato plants before HR symptoms (1 dai) and after HR symptoms developed (3 dai). Significant values (RLI > 1, and % Χ^2^ at least 10%) are in bold font and marked with an asterisk.

Sample	Parameters of Immunogold Labeling				
	*G_0_*	*Ge*	*RLI*	*Χ^2^ Value*	*Χ^2^ as %*
Immunogold Localization of RbohD in Potato Rywal (Less Resistant):					
**1. Mock-inoculated potato Rywal plants**					
cell wall	5	3	1.67	1.33	1.02
nucleus	4	3	1.33	0.33	0.25
endoplasmic reticulum (ER)	4	3	1.33	0.33	0.25
Golgi apparatus	12	2	6.00 *	50.00	38.17 *
chloroplasts	9	2	4.50 *	24.50	18.71 *
mitochondrion	1	7	0.14	5.14	3.93
vacuole	10	3	3.33 *	16.33	24.43 *
cytoplasm	2	4	0.50	1.00	0.76
vesicles	10	2	5.00 *	32.00	24.43 *
Column total				130.98	
**2. 1 day after PVY^NTN^-inoculation potato Rywal plants**					
cell wall	24	9	2.67 *	25.00	13.88 *
nucleus	20	6	3.33 *	32.67	18.14 *
endoplasmic reticulum (ER)	2	6	0.33	2.67	1.48
Golgi apparatus	10	2	5.00 *	32.00	17.77 *
chloroplasts	12	3	4.00 *	27.00	14.99 *
mitochondrion	14	3	4.67 *	40.33	22.39 *
vacuole	29	9	3.22 *	44.44	24.68 *
cytoplasm	6	4	1.50	1.00	0.56
vesicles	7	6	1.17	0.17	0.09
Column total				180.11	
**3. 3 days after PVY^NTN^-inoculation potato Rywal plants**					
cell wall	24	9	2.67	25.00	8.76
nucleus	8	6	1.33	0.67	0.23
endoplasmic reticulum (ER)	2	6	0.33	2.67	0.93
Golgi apparatus	10	2	5.00 *	32.00	11.22 *
chloroplasts	45	10	4.50 *	122.50	42.94 *
mitochondrion	24	5	4.80 *	72.20	25.31 *
vacuole	30	9	3.33 *	49.00	17.18 *
cytoplasm	9	4	2.25	6.25	2.19
vesicles	6	5	1.20	0.20	0.07
Column total				285.28	

**Table 3:** Quantification of immunogold labeling of RbohD by RLI and Χ^2^ tests in mock-inoculated and PVY^NTN^-inoculated Irys before systemic symptoms (7 dai) and after (21 dai) systemic necrosis developed. Significant values (RLI > 1, and % Χ^2^ at least 10%) are in bold font and marked with an asterisk.

Sample	Parameters of Immunogold Labeling				
	*G_0_*	*Ge*	*RLI*	*Χ* *^2^ Value*	*Χ* *^2^ as %*
Immunogold localization of RbohD in potato Irys (susceptible):					
**1. Mock-inoculated potato Irys plants**					
cell wall	5	1	5.00	16.00	9.49
nucleus	1	2	0.50	0.50	0.30
endoplasmic reticulum (ER)	2	6	0.33	2.67	1.58
Golgi apparatus	10	1	10.00 *	81.00	48.02 *
chloroplasts	7	1	7.00 *	36.00	21.34 *
mitochondrion	1	1	1.00	0.00	0.00
vacuole	1	2	0.50	0.50	0.30
cytoplasm	10	2	5.00 *	32.00	18.97 *
vesicles	1	2	0.50	0.50	0.30
Column total				168.67	
**2. 7 days after PVY^NTN^-inoculation potato Irys plants**					
cell wall	19	5	3.80	39.20	7.65
nucleus	1	2	0.50	0.50	0.10
endoplasmic reticulum (ER)	2	6	0.33	2.67	0.52
Golgi apparatus	10	1	10.00 *	81.00	15.80 *
chloroplasts	30	3	10.00 *	243.00	47.41 *
mitochondrion	25	5	5.00 *	80.00	15.61 *
vacuole	23	5	4.60 *	64.80	12.64 *
cytoplasm	5	3	1.67	1.33	0.26
vesicles	32	16	2.00	16.00	3.12
Column total				512.50	
**3. 21 days after PVY^NTN^ inoculation potato Irys plants**					
cell wall	15	5	3.00	20.00	4.60
nucleus	1	2	0.50	0.50	0.11
endoplasmic reticulum (ER)	1	5	0.20	3.20	0.74
Golgi apparatus	5	1	5.00	16.00	3.68
chloroplasts	22	2	11.00 *	200.00	45.99 *
mitochondrion	2	5	0.40	1.80	0.41
vacuole	10	2	5.00	32.00	7.36
cytoplasm	25	3	8.33 *	161.33	37.10 *
vesicles	12	5	2.40	9.80	2.25
Column total				434.83	
